# Workshops and Drink

**Published:** 1895-03-23

**Authors:** 


### The Hospital

An Institutional Journal of

The Medical Sciences and Hospital Administration, with

An Extra Nursing Supplement.

Vol. XVII.—No. 443.] [March 23, 1895.

### Workshops and Drink.

The Society for the Study of Inebriety accomplishes something, but it might by a little well-directed effort accomplish, we think, a good deal more. The paper on "Unwholesome Workshops and Drink," read at the last quarterly meeting by Dr. David Walsh, and discussed at some length by the members, well illustrates this proposition. It was a well-intentioned contribution to an important subject, and its main thesis, that workshops which are small and unhealthy are injurious to the men and women who work in them, and often help to drive them to drinking habits, is quite indisputable. So much we all believed before the subject was discussed by the Inebriety Society, and so much we all believe still. But the question in itself is much more important than this rather limited conception of it might lead one to suppose; and a discussion which should have brought out the full range and significance of the facts of the case might have illuminated and stimulated public opinion far and wide, and led to important and much-needed legislation.

Dr. Walsh devoted a considerable portion of a very brief paper to the setting forth of the chief causes of intemperance in general; he also compared the general mortality of country workers with that of town workers, and gave some scientific reasons of a general character why he considered it probable that certain classes of workshops tended to the production of drunkenness. The speakers who took part in the discussion agreed with propositions which were so obvious that disagreement was practically impossible; and there the matter ended. Now, we venture to think that a society with so considerable a reputation as that of the Society for the Study of Inebriety did not do itself justice by so perfunctory a consideration of a question which, in view of the many urgent politico economic problems connected with the present condition of the British working man is one of the most important political questions of the hour.

In the first place it ought to be demonstrated whether drunkenness is or is not actually a product of the modern workshop; and it should be demonstrated by facts and concrete evidence, not alone by arguments addressed to the understanding of that limited class who have had the advantage of an elementary scientific training. Next it ought to be shown, and still by undeniable facts, not by general reasons of an abstract or specialised character, what is the extent of the drunkenness, what is the approximate number of drunkards who can be proved to be annually manufactured by unhealthy workshops. Thus we should get a complete picture of the whole deadly circumstances of the case; and we should be able to go to the legislature and say, so many tens of thousands of our toiling industrial classes are made drunkards annually by unwholesome workshops, so many in consequence are annually consigned to premature graves; and so many tens of thousands of widows and orphans are thus thrown annually upon public charity or upon the rates. This ought not to be; it must not be. The State has the power to decree that the creators and the utilisers of factories and workshops shall make their factories and workshops healthy. They shall make them healthy, or they shall not make them or utilise them at all.

A task of this kind, we admit, would be gigantic. But just as gigantic as the task would be the service rendered to the public, and the honour gained by him who should undertake it and carry it to worthy completion. Facts accumulated, comparisons made, and conclusions reasoned out on adequate concrete data; these are what we need. Take, for example, a town like Leeds, containing every variety of factory or workshop. There you have iron industries which kill people by means of heat; cloth mills in which heat plays little or no part, but dust is a factor in the production of death; and an infinite variety of intervening industries. Round about Leeds are outlying villages, situated on hills, in valleys, or on sunny slopes, in which are carried on exactly the same classes of industry as are carried on in Leeds itself. But in the villages the factories and workshops are comparatively spacious and are surrounded on all sides by abundance of pure air and sunlight. In Leeds itself, on the other hand, all the industries are carried on under conditions of the most strictly limited space; and the surrounding atmosphere is choked with every conceivable impurity.

Here, then, we have the elements of a most effective and convincing comparison. Trades in the villages can be compared with the similar trades in the large town; large factories can be compared with small; workshops surrounded and sweetened by relatively pure country air can be compared with workshops in crowded and unwholesome town slums. If now we could show that a much larger proportion of men in the same trades become drunkards and die prematurely in Leeds than in the country villages round about that town; and if we could carry our comparisons to Manchester, Glasgow, Bristol, Birmingham, London, and throughout the country, furnishing always approximate figures as well as undeniable facts, we should present to the country and to the legislature a picture so striking, so convincing, and so deplorable that legislation of a remedial character would be entered upon by both political parties without a moment's unnecessary delay. The Study of Inebriety Society is no doubt primarily a medical society; but the "medicine" which, as a society, it studies—or should study—is medicine which is to a large extent of a social and a politico economic character. If the society would devote some real energy and intellectual capacity to this side of its work instead of employing all its opportunities on the endless repetition of a few physiological and pathological platitudes, with which everybody is familiar, and which nobody disputes, it might yet render important services to the public and the State, and justify those far seeing persons whose honour it was that they projected and established such a society.

